# Yield and Silage Quality of Winter Legume Cover Crop Mixtures Without Nitrogen Fertilization in Spring

**DOI:** 10.3390/plants14050726

**Published:** 2025-02-27

**Authors:** Marko Zupanič, Tomaž Žnidaršič, Miran Podvršnik, Vilma Sem, Boštjan Kristan, Ludvik Rihter, Branko Kramberger

**Affiliations:** 1 Faculty of Agriculture and Life Sciences, University of Maribor, Pivola 10, 2311 Hoče, Slovenia; 2 Agricultural Institute of Slovenia, Hacquetova ulica 17, 1000 Ljubljana, Slovenia; 3 Agricultural and Forestry Institute Maribor, Vinarska ulica 14, 2000 Maribor, Slovenia

**Keywords:** clover, grass, management practices, mixtures, pure stand, silage quality

## Abstract

A field experiment was conducted in two seasons (2019–2020 and 2020–2021) at three locations in Slovenia (Rogoza, Fala, and Brežice) to evaluate the yield and silage quality of winter cover crops (WCCs). The experiment included Italian ryegrass (IR) in pure stands, fertilized with nitrogen in spring, and mixtures of crimson clover (CRC), red clover (RC), and IR+CRC+RC without nitrogen fertilization in spring. The highest dry matter yield (DMY) was observed in IR+CRC+RC (4.98 t ha^−1^). For fresh feed, the CRC+RC treatment had significantly higher (*p* < 0.05) crude protein (208 g kg^−1^ DM), nitrate nitrogen (116.7 mg kg^−1^ DM), and buffering capacity (1290 mmol kg^−1^ DM) but significantly lower (*p* < 0.05) dry matter (128 g kg^−1^) and water-soluble carbohydrates (121 g kg^−1^ DM). For silage, the CRC+RC treatment had significantly lower (*p* < 0.05) dry matter (476 g kg^−1^ silage), metabolic energy (9.65 MJ kg^−1^ DM), net energy of lactation (5.77 MJ kg^−1^ DM), and neutral detergent fiber (375 g kg^−1^ DM) but higher ammonia nitrogen (66.5 g kg^−1^ of total nitrogen), crude protein (158 g kg^−1^ DM), and acid detergent fiber (279 g kg^−1^ DM). No significant differences (*p* > 0.05) were found among treatments for acetic, lactic, and butyric acid, crude fat, pH, and soil mineral nitrogen (N_min_). The results of the study show that the same or higher DMY and a comparable quality of highly wilted silage can be produced with mixed Italian ryegrass and clovers compared with those of Italian ryegrass in pure stands. The experiment aimed to determine whether clover-based mixtures can achieve comparable silage quality and dry matter yield without spring N fertilizers compared with those of pure stands of Italian ryegrass fertilized in spring.

## 1. Introduction

According to Stevens [1], the current global population is expected to exceed 9.7 billion by 2050. Hunter et al. [2] demonstrated that an increase of approximately 25–70% above current production levels may be sufficient to meet the 2050 crop demand. This demand for increased food production necessitates more amendments, both organic and inorganic, as agriculture contends with a food waste crisis where 1.3 billion tons of annual food production are wasted [3]. Agriculture is shifting towards more sustainable and environmentally friendly practices in response to the need for safer food and the increased awareness of the environmental and human health risks associated with the overuse of pesticides and fertilizers [4]. Synthetic nitrogen (N) fertilizers, introduced for agricultural use at the start of the 20th century [5], remain a crucial factor in achieving food security globally [1].

Agriculture is critical in reducing poverty by providing dependable food supplies and fostering economic growth in several countries, including temperate regions [6]. However, this sector faces mounting challenges, such as soil degradation, declining soil organic matter, soil erosion, and erratic climate change, all of which pose threats to agriculture due to various abiotic factors [7]. The intensification of crop production during the 20th century undoubtedly led to an increase in yield and helped prevent hunger.

However, it has also had negative environmental impacts, such as soil exhaustion, reduced fertility, soil salinization and erosion, environmental degradation, health hazards, and biodiversity loss [8]. The non-selective use of pesticides, unbalanced fertilization, and irrigation threaten the sustainability of agriculture [9]. In light of these facts, re-evaluating our conventional agricultural practices and adopting eco-friendly alternatives is imperative [10]. Additionally, the supply of N severely limits food production [11], and the industrial N supply is predominantly based on fossil fuels and has multiple environmental impacts [12]. Mineral fertilizer costs are increasing, and their excessive use can have adverse environmental impacts [12]. Therefore, incorporating legumes into cropping systems is a possible way to increase sustainability and N self-sufficiency. Legumes can fix atmospheric N, thereby supplying and retaining N in the soil [13]. Legume-fixed N_2_ is a traditional alternative to mineral N fertilization in agricultural systems, with N fixation rates varying from 50 to 200 kg N ha^−1^ yr^−1^ [14] depending on various factors, such as existing soil mineral N, legume species, weather, and harvest management [15].

The use of conventional methods, such as mineral fertilizers, to achieve stable and high crop yields has become limited because they contribute to climate change [16]. Therefore, alternative approaches that maintain biodiversity and minimize environmental impacts must be developed. Mixed cropping, which involves the cultivation of multiple crops in the same field, has been proposed as a solution. Legume/non-legume species mixtures have been shown to increase per-area production and profitability while maintaining high-yield stability with little or no external inputs [17,18,19,20].

Mixtures of legumes and non-legumes have been widely used in mixed sowing systems to improve productivity and stability [21]. Legumes in mixtures improve the nutritional quality of the forage produced. Studies have reported better digestibility and higher crude protein (CP) content in grass–clover mixtures than in grass monocultures [22] and improved neutral detergent fiber (NDF) concentration and digestibility when, for example, alfalfa *Medicago sativa* was added to a grass mixture [23]. Adding red clover *Trifolium pratense* to a mixture has been shown to increase milk production and quality in dairy cows [24]. While legumes fix N biologically in temperate climates, they may not produce high biomass. In contrast, grasses and crucifers are often used as cover crops [25]. However, grasses can produce biomass with a wider C:N ratio, resulting in fewer benefits for subsequent crops [26], especially when the entire plant biomass yield is plowed down. Therefore, combining legumes and grasses is beneficial, as it produces sufficient biomass of good quality [18,27,28,29]. In some European countries, cover crop mixtures have been promoted because of their greater ecological services compared with those of pure stands [30,31], compensatory growth responses [32], and reduced risk of cover crop failure under adverse weather conditions [33]. Mixed stands of cover crops comprising highly productive species are more beneficial than pure stands of the most productive species in terms of biomass production and N uptake [34], which can also be useful when using aboveground biomass for silage production.

Legume–grass mixtures are highly suitable for producing high-quality silage for animal feed. Silage quality is influenced by various factors, including plant species, agro-technical practices, preparation time, applied inoculants (such as seeds of legumes infected with N-fixing bacteria), and the stage of plant development when used for silage preparation. Legumes are an important source of protein, but due to their high buffering capacity (BC) and low soluble sugar content, they are difficult to ensile. To overcome this issue, the authors suggested mixing legumes with grasses to improve their fermentation properties [35].

The biomass yield from grass–legume mixtures is often equal to or greater than that from component monocultures [36,37], suggesting that N functional diversity may lead to transgressive overyielding by cover crop mixtures. After comparing mixtures of red clover and ryegrass silages, Moorby et al. [38] concluded that the optimal milk yields were achieved with a ratio of 66% red clover silage in the forage component of the diet. Halmemies-Beauchet-Filleau et al. [39] also found increased feed intake and milk yields from feeding mixtures of grass and red clover compared with those from feeding grass alone.

This study aimed to demonstrate that at the farm production level, it is possible to achieve at least an equivalent amount of high-quality silage and dry matter yield in mixtures of clovers and clovers with ryegrass without using N fertilizers in spring compared with those of pure stands of Italian ryegrass fertilized in spring.

## 2. Results

The presented data show the averages of two years and three locations. The average initial soil mineral nitrogen (N_min_) content before the start of the experiment was 60.8 kg ha^−1^. The N_min_ decreased from sowing until May of the following year. No significant differences (*p* > 0.05) in N_min_ were observed among the treatments in November and May. The DMY was significantly higher (*p* < 0.05) in the treatment with IR and in the treatment with IR+CRC+RC (4.98 and 4.87 t ha^−1^, respectively) than in the CRC+RC treatment (4.25 t ha^−1^) (Table 1).

Before wilting and ensiling, the DM content of fresh feed in the IR and IR+CRC+RC treatments (173 and 170 g kg^−1^, respectively) was significantly higher (*p* < 0.05) than that in the CRC+RC treatment (128 g kg^−1^). The CP content in the IR and IR+CRC+RC treatments (113 and 117 g kg^−1^ DM, respectively) was significantly lower (*p* < 0.05) than that in the CRC+RC treatment (208 g kg^−1^ DM). The water-soluble carbohydrate (WSC) content in CRC+RC (121 g kg^−1^ DM) was significantly lower (*p* < 0.05) than that in IR and IR+CRC+RC (271 and 275 g kg^−1^ DM, respectively). The NO_3_-N content was significantly lower (*p* < 0.05) in IR than in CRC+RC (21.3 vs. 116.7 mg kg^−1^ DM). Conversely, IR and IR+CRC+RC did not differ significantly (*p* > 0.05). The BC in CRC+RC (1290 mmol kg^−1^ DM) was significantly higher (*p* < 0.05) than that in IR and IR+CRC+RC (818 and 917 mmol kg^−1^ DM, respectively) (Table 2).

The silage DM content in IR and IR+CRC+RC (588 and 562 g kg^−1^ silage, respectively) was significantly higher (*p* < 0.05) compared with that in CRC+RC (476 g kg^−1^ silage). No significant differences (*p* > 0.05) in pH, lactic acid (LA), acetic acid (AA), and butyric acid (BA) were observed. The NH_3_-N content in CRC+RC (66.5 g kg^−1^ of TN) was significantly higher (*p* < 0.05) than that in IR and IR+CRC+RC (34.7 and 40.3 g kg^−1^ of TN, respectively) (Table 3).

Silage metabolizable energy (ME), net energy of lactation (NEL), CP, and NDF significantly differed (*p* < 0.05) between IR and CRC+RC, as well as between CRC+RC and IR+CRC+RC. The ME content of IR and IR+CRC+RC (10.4 MJ kg^−1^ DM) did not significantly differ but was significantly higher than that in CRC+RC (9.65 MJ kg^−1^ DM). The NEL showed no significant difference between IR and IR+CRC+RC (6.26 and 6.27 MJ kg^−1^ DM, respectively); however, it was significantly higher than that in CRC+RC (5.77 MJ kg^−1^ DM). Likewise, CP content did not significantly differ (*p* > 0.05) between IR and IR+CRC+RC (112 and 113 g kg^−1^ DM, respectively) but was significantly lower (*p* < 0.05) than that in CRC+RC (158 g kg^−1^ DM) (Table 4).

Silage NDF content exhibited no significant differences between IR and IR+CRC+RC (463 and 429 g kg^−1^ DM, respectively) but was markedly higher compared with that in CRC+RC (375 g kg^−1^ DM). The ADF content did not significantly differ between IR and CRC+RC (246 and 279 g kg^−1^ DM, significantly). However, in CRC+RC, it was significantly higher than that in IR+CRC+RC (241 g kg^−1^ DM). ADF significantly differed (*p* < 0.05) only between CRC+RC (279 g kg^−1^ DM) and IR+CRC+RD (241 g kg^−1^ DM). No significant differences (*p* > 0.05) were observed for CFA. Higher ME, NEL, NDF, and CFA were achieved in IR and IR+CRC+RC, but CP and ADF were lower. No differences in the studied parameters were found between the IR and IR+CRC+RC (Table 4).

## 3. Discussion

In designing the study with WCCs, we aimed to investigate whether, under production conditions, it is possible to exploit the potential advantages of a mixture of Italian ryegrass and clovers without N fertilization in spring for silage compared with a pure stand of Italian ryegrass fertilized in spring. Clovers often have the advantage of containing more CP than Italian ryegrass [40], whereas Italian ryegrass contains more WSCs [41,42] and is easier to ensile [41]. Legume crops are more difficult to ensile due to their higher BC and lower WSC content [43]. This was also the case in our study, and it resulted in some silage fermentation parameters (Table 2). Energy values are generally higher in Italian ryegrass than those in legumes [44], as corroborated by our research (Table 4).

Before the start of the experiments, soil N_min_ content was 60.8 kg ha^−1^. In November of the current year and May of the following year, no differences were observed in soil N_min_ content among treatments (Table 1), despite the application of 70 kg N ha^−1^ to a pure Italian ryegrass stand in spring. When comparing the initial soil N_min_ content with the levels in November of the current year and May of the following year, a notable reduction in N levels was observed, suggesting effective N uptake by the crops in all treatments (Table 1). N content in the aboveground DMY was 88.04 kg ha^−1^ for IR, 141.4 kg ha^−1^ for CRC+RC, and 93.2 kg ha^−1^ for IR+CRC+RC. Similar spring soil N_min_ contents in N-fertilized Italian ryegrass compared with those in the other non-N-fertilized treatments can be explained by intensive N uptake by ryegrasses. This is consistent with Kramberger et al. [45,46], who found that Italian ryegrass had lower soil N_min_ contents in spring than those in clovers. Similarly, Sarunaite et al. [47] also observed lower soil N_min_ content in spring for perennial ryegrass compared with that in red clover in both years of their experiment.

N uptake in the unfertilized treatments can be attributed to symbiotic N fixation. Including a leguminous species in a mixture can promote the growth of adjacent plants by enhancing N availability through reduced mineral N uptake from the soil by the legume. Synergistic effects within mixtures occur because legumes and grasses have complementary resource utilization patterns. When legumes are grown in combination with grasses, they often obtain a larger portion of their N through biological N fixation than that under monoculture settings [48]. This ability of legumes to biologically fix N allows grasses to accumulate higher levels of tissue N in mixed plantings than in monoculture scenarios [36]. Biologically fixed N may even be transferred from legumes to companion grasses within mixtures, either directly through mycorrhizal hyphae or indirectly through the decomposition of organic materials derived from the legume [49]. Furthermore, grasses and legumes exhibit differences in their aboveground architecture, enabling mixed plantings to capture light more effectively than monocultures [50].

The aboveground DMY of the IR treatment (4.87 t ha^−1^) was similar to that of the IR+CRC+RC treatment (4.98 t ha^−1^), despite spring N fertilization of the ryegrass. In IR+CRC+RC, the DMY was higher compared with that in the CRC+RC treatment (Table 1), indicating positive complementary effects between legumes and grasses. Clovers in the mixture were fixing N from the air, which was utilized for plant growth. Ćupina et al. [51] also observed similar results, noting higher yields in mixtures compared with those in pure stands. The aforementioned authors conducted an experiment in Banja Luka in 2012 and 2013, where a mixture of Italian ryegrass and red clover achieved a higher DMY at the first cut compared with pure stands. They also conducted a similar experiment in Pristina, where a mixture of Italian ryegrass and red clover similarly achieved higher DMY at the first cut in 2013, 2014, and 2015. In the present study, we also achieved the highest DMY in the IR+CRC+RC treatment (Table 1). Several other studies have also reported higher aboveground mass yields in mixed than in pure stands [27,29,52,53].

The current study aimed to quantify the effects of WCC treatments on silage nutritive value and fermentation characteristics. One of the most important factors influencing the fermentation behavior of silage is the DM content of the ensiled material [54]. In our experiment, under practical conditions, the IR treatment showed a higher drying rate than the IR+CRC+RC and CRC+RC treatments (Table 2). Researchers have noted that the plant species affects drying rates [55,56]. Typically, grass species tend to dry faster than legume species [57]. Variations in drying rates among species are primarily due to variations in the physical attributes of plants. Species with the highest ratios of surface area to dry weight typically exhibit the fastest drying rates [58]. Consequently, leaves tend to dry more rapidly than stems. Leaves naturally serve as the main pathway for moisture loss in plants. When all other factors are identical, forage with a higher leaf density dries faster than that with fewer leaves [55].

For successful LA fermentation, feed must contain sufficient WSCs while maintaining a moderate BC. WSC and BC factors considerably affect the fermentation process and reduce the risk of silage spoilage [59]. In our study, the highest WSC content was achieved in the IR+CRC+RC treatment, followed by the IR treatment, while the lowest was observed in the CRC+RC treatment (Table 4). Similar results were reported by Moloney et al. [41]. The highest BC content in the current study was found in the CRC+RC treatment (1290 mmol kg^−1^ DM), while the lowest was in the IR treatment (818 mmol kg^−1^ DM) (Table 2). This finding aligns with the study by Moloney et al., who also reported that clovers have a higher BC than that of grasses [41].

For LA fermentation, the moisture content of the feed is crucial, as sugars concentrate during wilting under favorable conditions and because the growth of clostridia is directly related to water activity [60]. In the conducted experiments, the moisture content of the yield before wilting was appropriate (Table 2), promoting intensive fermentation in the silages across all treatments (Table 3). The DM content in silage was lowest in the CRC+RC treatment among all treatments (476 g kg^−1^). Favorable LA fermentation was observed in all silages made from Italian ryegrass and from mixtures. AA levels were low, and BA was minimally present in the silage of the IR and CRC+RC treatments, whereas BA was not present in the silage of the IR+CRC+RD treatment (Table 3). Silages with desirable LA fermentation were characterized by low pH and AA, BA, and NH_3_-N contents [61]. Here, we achieved higher LA and AA contents in the IR treatment compared with those in the other treatments (Table 3). Moloney et al. [41] achieved a higher LA content with a mixture of Italian ryegrass and red clover compared with that in pure stands of red clover and Italian ryegrass. Similar results were obtained in an experiment by Li et al. [62], where a mixture of Italian ryegrass and burr clover resulted in higher AA and lower LA and BA contents compared with those in pure stands. Furthermore, in an experiment with a mixture of grasses and clovers, Damborg et al. [63] achieved lower LA (20.9 g kg^−1^ DM), AA (2.85 g kg^−1^ DM), and DM (514 g DM kg^−1^) contents and higher BA contents (0.09 g kg^−1^ DM) than those in the present study (Table 3). Kung et al. [64] provided recommended concentrations for LA, BA, and NH_3_-N in legume silage containing <30–35% DM as follows: 6–8% for LA, <0.5% for BA, and 10–15% for the NH_3_-N of total N. For legume silage containing 45–55% DM, they recommended the following concentrations: 2–4% for LA, 0% for BA, and <12% for the NH_3_-N of total N. For grass silage containing 25–35% DM, they recommended the following concentrations: 6–10% for LA, <0.5–1% for BA, and 8–12% for the NH_3_-N of total N.

In the current study, the NH_3_-N content in the silage of the mixture of CRC+RC was higher compared with that in the silages of the other treatments (Table 3). This suggests that ensiling clovers is somewhat more challenging. Li et al. [62] reported that an Italian ryegrass and burr clover (in a 50:50 ratio) mixture exhibited a higher NH_3_-N content than that in Italian ryegrass pure stands and lower NH_3_-N content than that in the pure stands of burr clover (Table 3). We found that clover silages exhibited slightly poorer fermentation characteristics compared with those from Italian ryegrass and its mixture with clovers. These contained slightly higher levels of NH_3_-N and BA (Table 3), indicating slightly more extensive activity of undesirable clostridia in the silage. However, from a practical perspective, these relatively small differences were not significant.

In the present study, clover silage contained a lower NEL content compared with that in the other treatments (Table 4), which may be due to a reduced NEL during the preparation of the feed for ensiling [65,66], including its contamination with soil and a decrease during silage fermentation. We found a favorable effect of mixing Italian ryegrass and clovers in improving the energy value. The energy value of the IR+CRC+RC treatment was higher than that of the CRC+RC treatment (Table 4). These results are expected, as Italian ryegrass exhibits an excellent net energy value [67]. We would have expected a value intermediate between the IR and CRC+RC treatments, but the value was at the level of the IR treatment. The IR+CRC+RC treatment met expectations regarding its net energy value, since the NEL content was on par with that of the IR treatment (Table 4). Similar results are also reported by Homolka et al. [68].

The highest CP content was observed in the CRC+RC treatment (Table 4). The lower CP content in IR+CRC+RC is somewhat surprising, but it can be explained. CP content in Italian ryegrass significantly increases with N fertilization [69,70,71,72]. The IR+CRC+RC treatment was not fertilized with N, resulting in the IR in the mixture containing less protein (83.1 g kg^−1^ DM—data not presented) than the IR in the pure stand (113 g kg^−1^ DM) (Table 2). CP contents similar to those achieved in our study with the mixture (Table 4) were also reported by Merkevičiūtė-Venslovė et al. [73], whereas our CP content was exceeded by that reported by Li et al. [62] and Thers et al. [74]. The similar CP content in the IR+CRC+RC and IR treatments indicates that clovers in the mixture did not succeed in increasing the CP in the silage; however, it seems that N from clovers was sufficient only for increasing the DMY of the mixture.

In the current study, the clovers had a lower NDF and a higher ADF content than those of the grasses (Table 4). Legumes reportedly have lower NDF and ADF contents than those of the grasses [75,76,77]. Lower ADF values indicate higher energy content and improved digestibility [78]. Egan et al. [79] reported that grass–clover mixtures had lower NDF and ADF contents than those in pure stands of grass, as we also found in our study (Table 4). Li et al. [62] indicated that an Italian ryegrass and burr clover (in a 50:50 ratio) mixture exhibited higher and lower NDF and ADF contents than those in pure stands of Italian ryegrass and burr clover, respectively. In their study, mixtures with annual ryegrass and burr clover (in a 50:50 ratio) had higher ADF and NDF contents than those in our experiment (Table 4).

## 4. Materials and Methods

### 4.1. Experimental Site, Treatments, and Crop Management

Field experiments were conducted in two winter growing seasons (2019–20 and 2020–21) at three different locations in Slovenia (Rogoza: 46°29′59.15″ N, 15°40′49.75″ E, 266 MASL; Fala: 46°32′43.35″ N, 15°27′15.67″ E, 306 MASL; and Brežice: 45°54′24.17″ N, 15°35′30.00″ E, 162 MASL). Three winter cover crops (WCCs) were used in this experiment, namely Italian ryegrass (*Lolium multiflorum* L., cultivar Melquatro), crimson clover (*Trifolium incarnatum* L., cultivar Heusers ostsaat), and red clover (*Trifolium pratense* L., cultivar global) (Table 5). A randomized complete block design was used for all sites. Different fields were used in the 2 years to avoid the cumulative effects of growing the same mixture at the same site in subsequent years. Sand, silt, and clay contents in soil samples were determined using the sieving and sedimentation method [80]. Based on the proportion of individual size fractions, we determined the soil texture using the texture triangle [81]. The soil texture was clay in Rogoza and silty clay in Brežice. The soil organic matter content was highest in Brežice (2.2%) and lowest in Fala (1.7%) in the first year of the experiment. In the second year, the organic matter content was again highest in Brežice (2.8%) and lowest in Rogoza (1.5%) (Table 6).

The seedbed was prepared at a depth of 8 cm using a power harrow. Before sowing the WCCs, we fertilized the fields with 50 kg of N, 70 kg of P_2_O_5_, and 120 kg of K_2_O ha^−1^. The WCCs were sown at the end of August and harvested the following year in May. In the spring of the following year, we fertilized only Italian ryegrass in a pure stand with 70 kg N ha^−1^ as potassium ammonium nitrate (27% N). The sowing and harvesting dates of the WCCs varied across site years because of variations in the harvest date of the preceding cash crop and rainfall at the site. The details for all sites, including field operations and weather conditions, are summarized in Table 6.

Six random soil samples (0–30 and 30–60 cm) were collected from each plot before cover crop sowing at the end of November and at the beginning of May. The total N_min_ in the samples was determined using calcium chloride extraction (CaCl_2_) [82]. The AL method according to Egnér et al. [83] was used to determine the P_2_O_5_ and K_2_O concentrations. Organic matter was determined according to the method of Walkley and Black [84] (Table 6).

To determine the DMY of the WCCs, we collected six samples of aboveground biomass within each treatment, covering an area of 0.25 m^2^ and using electric shears 5 cm above the ground, and we then dried them (60° C for 48 h). Each block contained three treatments. The size of each treatment was 3000 m^2^. The entire yield of WCCs after partial drying on the ground (wilting) was ensiled in bales. One month after ensiling, in both years and at all locations, we took two samples from the silage bales using a separate probe for each treatment. We dried and ground a portion of the sample, while another portion was frozen for the determination of acids (acetic, lactic, butyric, and the corresponding pH values). The samples from both years were analyzed separately (Table 5). The concentration of acids in the silages was measured using gas chromatography, following the method described by Holdeman and Moore [85]. The ammonia content in the silage was determined using the Kjeldahl method [86]. The total N in the soil was determined by the Kjeldahl method of Keeney and Nelson [82] (Table 1). The parameters of the silage samples were analyzed using near-infrared reflectance spectroscopy (NIRS).

The concentrations of hygroscopic moisture, CP (g kg^−1^ DM), crude ash (CA) (g kg^−1^ DM), CFA (g kg^−1^ DM), NDF (g kg^−1^ DM), ADF (g kg^−1^ DM), WSCs (g kg^−1^ DM), and gas produced (mL (200 mg)^−1^ DM)) during the incubation of the samples with rumen fluid in vitro (GP) were determined using NIRS according to the principles described by Žnidaršič et al. [87]. Calibration equations were developed using a partial least squares regression technique using information from a large number of grassland forage samples, including the samples from the present study (870 samples for CP and CA, 259 for NDF, 295 for ADF, 421 for WSC, and 650 for GP). Metabolizable energy (ME) and net energy of lactation (NEL) were calculated based on chemical composition and in vitro GP. Specific equations for grasses [88] and legumes [89] were applied for IR and CRC+RC, respectively. For IR+CRC+RC, the average value of the two equations was employed.

### 4.2. Statistical Analyses

Linear mixed-effects models (LMERs) were used to analyze the effects of WCCs (fixed factors) on DM, DMY, pH, N_min_, LA, AA, BA, NH_3_-N, BC, NO_3_-N, ME, NEL, WSC, NDF, ADF, and CFA (dependent variables). Year and location variables were included in the model as random factors to control for variance associated with measures taken in the same year or location. Because a visual inspection of the residual plots for the fitted models revealed no visible deviations from homoscedasticity or normality, no data transformation method was used. Fisher’s least significant difference (LSD) test was used to detect significant differences between all pairs of treatments. Statistical analyses were performed using R version 4.2.2 [90] (R Foundation for Statistical Computing, Vienna, Austria). LMERs were fitted using the lmer function from the lme4 library [91], and *p*-values for fixed effects were obtained using Satterthwaite approximations implemented in the lmerTest library [92]. Multiple comparisons were performed using Fisher’s least significant difference test using the library glht [93].

## 5. Conclusions

Sowing WCCs after harvesting the main crop, especially in mixtures, has become a crucial practice in temperate agriculture for producing high-quality bulk forage.

The important conclusions of the present study are as follows:

1. We achieved comparable yields during the spring period with grass–legume mixtures without spring N fertilization compared with N-fertilized pure stands of Italian ryegrass in spring.
2. The grass–legume mixture is more suitable for ensilage compared with clovers (higher WSCs and lower BC), but high-quality silage can also be prepared from legumes.
3. We achieved a comparable net energy value with the grass–legume mixture relative to that of Italian ryegrass and better than that of the legume mixture.
4. Contrary to expectations, we did not succeed in increasing the protein content in the silage comprising the grass–legume mixture without N fertilization in spring compared with pure stands of Italian ryegrass fertilized with N in spring.

In conclusion, this experiment demonstrated that comparable or even higher silage yields and quality could be achieved in spring with mixtures containing legumes that are not fertilized with N, compared with those of nitrogen-fertilized pure stands of Italian ryegrass. This approach saves time and money and can positively affect the environment. The results are important for farms with large livestock populations and high demands for quality feed. Mixtures offer the advantage of promoting species diversity and reducing N use while also increasing competition for nutrients, growing space, and water. However, no significant benefits were observed in species mixtures.

## Figures and Tables

**Table 1:** Soil mineral nitrogen and the effect of treatments on dry matter yield.

Parameter	Treatment		
	IR	CRC+RC	IR+CRC+RC
N_min_ in November (kg ha^−1^)	26.6	28.3	26.8
N_min_ in May (kg ha^−1^)	12.0	16.1	15.1
DMY (t ha^−1^)	4.87 ^a^	4.25 ^b^	4.98 ^a^

^a^, ^b^ Means followed by different superscript letters in the same row indicate significant differences between treatments (*p* < 0.05). DMY, dry matter yield; N_min_, mineral nitrogen; IR, Italian ryegrass; CRC, crimson clover; RC, red clover.

**Table 2:** Effect of treatments on different parameters before wilting and ensiling.

Parameter	Treatment		
	IR	CRC+RC	IR+CRC+RC
DM (g kg^−1^)	173 ^a^	128 ^b^	170 ^a^
CP (g kg^−1^ DM)	113 ^a^	208 ^b^	117 ^a^
WSC (g kg^−1^ DM)	271 ^a^	121 ^b^	275 ^a^
NO_3_-N (mg kg^−1^ DM)	21.3 ^a^	116.7 ^b^	39.7 ^ab^
BC (mmol kg^−1^ DM)	818 ^a^	1290 ^b^	917 ^a^

^a^, ^b^ Means followed by different superscript letters in the same row indicate significant differences between treatments (*p* < 0.05). DM, dry matter; CP, crude protein; WSC, water-soluble carbohydrate; BC, buffering capacity; IR, Italian ryegrass; CRC, crimson clover; RC, red clover.

**Table 3:** Effect of treatments on fermentation parameters in silage.

Parameter	Treatment		
	IR	CRC+RC	IR+CRC+RC
DM (g kg^−1^ silage)	588 ^a^	476 ^b^	562 ^a^
pH (-)	4.78	4.69	4.86
LA (g kg^−1^ DM)	30.4	26.8	26.4
AA (g kg^−1^ DM)	5.34	4.76	4.59
BA (g kg^−1^ DM)	0.22	0.75	0
NH_3_-N (g kg^−1^ of TN)	34.7 ^a^	66.5 ^b^	40.3 ^a^

^a^, ^b^ Means followed by different superscript letters in the same row indicate significant differences between treatments (*p* < 0.05). DM, dry matter; LA, lactic acid; AA, acetic acid; BA, butyric acid; TN, total nitrogen; IR, Italian ryegrass; CRC, crimson clover; RC, red clover.

**Table 4:** Effect of treatments on nutritional parameters in silage.

Parameter	Treatment		
	IR	CRC+RC	IR+CRC+RC
ME (MJ kg^−1^ DM)	10.4 ^a^	9.65 ^b^	10.4 ^a^
NEL (MJ kg^−1^ DM)	6.26 ^a^	5.77 ^b^	6.27 ^a^
CP (g kg^−1^ DM)	112 ^a^	158 ^b^	113 ^a^
NDF (g kg^−1^ DM)	463 ^a^	375 ^b^	429 ^a^
ADF (g kg^−1^ DM)	246 ^ab^	279 ^a^	241 ^b^
CFA (g kg^−1^ DM)	19	15.7	16.6

^a^, ^b^ Means followed by different superscript letters in the same row indicate significant differences between treatments (*p* < 0.05). ME, metabolizable energy; NEL, net energy of lactation; CP, crude protein; NDF, neutral detergent fiber; ADF, acid detergent fiber; CFA, crude fat; IR, Italian ryegrass; CRC, crimson clover; RC, red clover.

**Table 5:** Composition of the seed mixtures.

Treatment	Percentage of Seed in the Mixture (%)			Seeding Rate (kg ha^−1^)		
	IR	CRC	RC	IR	CRC	RC
1. IR	100			40		
2. CRC+RC		50	50		15	12.5
3. IR+CRC+RC	50	25	25	20	7.5	6.25

Percentages and amounts are based on the seeding rate of each species in a pure stand. IR, Italian ryegrass; CRC, crimson clover; RC, red clover.

**Table 6:** Site descriptions, field operations, and prevailing weather conditions in 2019–20 and 2020–21 at three sites, namely Rogoza, Fala, and Brežice.

Site Characteristics	2019–20 2020–21					
	Rogoza	Fala	Brežice	Rogoza	Fala	Brežice
Sand (%)	33.8	53.4	23.5	31.2	28.8	21.4
Silt (%)	47.5	30.4	55.8	42.4	45.6	57.5
Clay (%)	18.7	16.2	20.7	26.4	25.6	21.1
Soil texture	clay	sandy clay	silty clay	clay	cay	silty clay
Soil organic matter (%)	1.8	1.7	2.2	1.5	2.2	2.8
Soil pH (CaCl_2_)	6.2	6.3	5.3	5.3	6.4	5.8
P_2_O_5_ (mg/100 g soil)	16.1	14.7	9.2	13.0	15.2	10.2
K_2_O (mg/100 g soil)	20.6	16.9	20.2	18.7	14.5	19.4
Previous crop	oilseed rape	barley	barley	barley	wheat	wheat
Sowing date	27 August	28 August	29 August	26 August	28 August	29 August
Fertilizer before sowing WCCs (50 kg N; 70 kg P_2_O_5_; 120 kg K_2_O ha^−1^)	the entire experimental area			the entire experimental area		
Nitrogen application in spring (kg N ha^−1^)	70 *	70 *	70 *	70 *	70 *	70 *
Harvesting date	6 May	3 May	2 May	10 May	8 May	9 May
Plot size (m^2^)	3000	3000	3000	3000	3000	3000
Sum of precipitation during the growth period (from the end of August to the beginning of May) (mm)	470	498	648	562	569	680

* At all three locations, only the Italian ryegrass treatment in a pure stand was fertilized with N. WCCs, winter cover crops.

## Data Availability

Data will be made available upon request.

## Abbreviations

The following abbreviations are used in this manuscript:

AA	Acetic acid
ADF	Acid detergent fiber
BA	Butyric acid
BC	Buffering capacity
CFA	Crude fat
CP	Crude protein
CRC	Crimson clover
DM	Dry matter
DMY	Dry matter yield
IR	Italian ryegrass
LA	Lactic acid
LSD	Least significant difference
ME	Metabolizable energy
N	Nitrogen
N_min_	Soil mineral nitrogen
NDF	Neutral detergent fiber
NEL	Net energy of lactation
NIRS	Near-infrared reflectance spectroscopy
RC	Red clover
WCCs	Winter cover crops
WSC	Water-soluble carbohydrate
